# PKA and Epac cooperate to augment bradykinin-induced interleukin-8 release from human airway smooth muscle cells

**DOI:** 10.1186/1465-9921-10-88

**Published:** 2009-09-29

**Authors:** Sara S Roscioni, Loes EM Kistemaker, Mark H Menzen, Carolina RS Elzinga, Reinoud Gosens, Andrew J Halayko, Herman Meurs, Martina Schmidt

**Affiliations:** 1 Department of Molecular Pharmacology, University of Groningen, Groningen, The Netherlands; 2 Departments of Physiology and Internal Medicine, University of Manitoba, Winnipeg, Manitoba, Canada

## Abstract

**Background:**

Airway smooth muscle contributes to the pathogenesis of pulmonary diseases by secreting inflammatory mediators such as interleukin-8 (IL-8). IL-8 production is in part regulated via activation of G_q_-and G_s_-coupled receptors. Here we study the role of the cyclic AMP (cAMP) effectors protein kinase A (PKA) and exchange proteins directly activated by cAMP (Epac1 and Epac2) in the bradykinin-induced IL-8 release from a human airway smooth muscle cell line and the underlying molecular mechanisms of this response.

**Methods:**

IL-8 release was assessed via ELISA under basal condition and after stimulation with bradykinin alone or in combination with fenoterol, the Epac activators 8-pCPT-2'-O-Me-cAMP and Sp-8-pCPT-2'-O-Me-cAMPS, the PKA activator 6-Bnz-cAMP and the cGMP analog 8-pCPT-2'-O-Me-cGMP. Where indicated, cells were pre-incubated with the pharmacological inhibitors *Clostridium difficile *toxin B-1470 (GTPases), U0126 (extracellular signal-regulated kinases ERK1/2) and Rp-8-CPT-cAMPS (PKA). The specificity of the cyclic nucleotide analogs was confirmed by measuring phosphorylation of the PKA substrate vasodilator-stimulated phosphoprotein. GTP-loading of Rap1 and Rap2 was evaluated via pull-down technique. Expression of Rap1, Rap2, Epac1 and Epac2 was assessed via western blot. Downregulation of Epac protein expression was achieved by siRNA. Unpaired or paired two-tailed Student's t test was used.

**Results:**

The β_2_-agonist fenoterol augmented release of IL-8 by bradykinin. The PKA activator 6-Bnz-cAMP and the Epac activator 8-pCPT-2'-*O*-Me-cAMP significantly increased bradykinin-induced IL-8 release. The hydrolysis-resistant Epac activator Sp-8-pCPT-2'-*O*-Me-cAMPS mimicked the effects of 8-pCPT-2'-*O*-Me-cAMP, whereas the negative control 8-pCPT-2'-*O*-Me-cGMP did not. Fenoterol, forskolin and 6-Bnz-cAMP induced VASP phosphorylation, which was diminished by the PKA inhibitor Rp-8-CPT-cAMPS. 6-Bnz-cAMP and 8-pCPT-2'-*O*-Me-cAMP induced GTP-loading of Rap1, but not of Rap2. Treatment of the cells with toxin B-1470 and U0126 significantly reduced bradykinin-induced IL-8 release alone or in combination with the activators of PKA and Epac. Interestingly, inhibition of PKA by Rp-8-CPT-cAMPS and silencing of Epac1 and Epac2 expression by specific siRNAs largely decreased activation of Rap1 and the augmentation of bradykinin-induced IL-8 release by both PKA and Epac.

**Conclusion:**

Collectively, our data suggest that PKA, Epac1 and Epac2 act in concert to modulate inflammatory properties of airway smooth muscle via signaling to the Ras-like GTPase Rap1 and to ERK1/2.

## Background

Asthma and chronic obstructive pulmonary disease (COPD) are chronic inflammatory diseases characterized by structural and functional changes of the airways [1,2]. The underlying pathogenic processes of asthma and COPD include the production and release of chemokines and cytokines by inflammatory and structural cells [3]. Airway smooth muscle cells have recognized as immunomodulatory cells able to synthesize multiple inflammatory mediators such as cytokines, including interleukin-8 (IL-8) [4-6].

IL-8 represents one of the best characterized members of the family of chemokines known to attract and activate leukocytes and plays a major role in the initiation and maintenance of inflammatory responses [7]. In particular, IL-8 is a potent chemoattractant for neutrophils and eosinophils [8,9], that have been implicated in inflammatory airway diseases [10]. Indeed, enhanced IL-8 has been detected in blood and bronchial mucosa [11] and in bronchial epithelial cells of patients with asthma [12], in bronchoalveolar lavage fluid (BALF) of asthmatic and chronic bronchitis patients [13], in BALF and sputum from patients with COPD [14,15]. IL-8 levels correlate with the number of airway neutrophils, which are strongly associated with severe asthma and are increased during acute exacerbations of chronic bronchitis [16]. Airway smooth muscle are a rich source of IL-8 [6]. The gene expression of IL-8 is tightly regulated by inflammatory and pro-contractile agonists [6,17,18] acting on the large superfamily of G-protein-coupled receptors (GPCRs).

Bradykinin is a pluripotent nonapeptide generated by plasma and tissue kallikreins, and is upregulated in patients with asthma [19]. It has been reported that bradykinin stimulates the expression of IL-8 in human lung fibroblasts and airway smooth muscle [6,18]. This response is coupled to activation of extracellular signal-regulated protein kinases 1 and 2 (ERK1/2) [18,20] and appears to involve cyclooxygenase-dependent and -independent signals [6,21].

G_s_-protein-coupled receptor activation (e.g. β_2_-adrenergic or prostanoid receptors) modulates the release of cytokines from airway cells [6], probably via activation of adenylyl cyclase and subsequent increase in intracellular cyclic AMP (cAMP). Importantly, a synergism between bradykinin and the cAMP-elevating agents salmeterol and prostaglandin E_2 _(PGE_2_) has been reported at the level of IL-6 production from airway smooth muscle [22]. Although these studies clearly indicate a role for cAMP in pro-inflammatory cytokine production, the engagement of distinct cAMP-regulated effectors has not been yet addressed in the airways. Given the importance of the bradykinin- and the cAMP-driven pathways in both the pathophysiology and the treatment of pulmonary diseases, insights into the cellular mechanisms of their interaction are warranted.

Indeed, increasing evidence suggests that cAMP actively regulates transcription and gene expression events in several airway cells [23,24], and that such mechanism may regulate local cytokine production in human airway smooth muscle [21]. Until recently, intracellular effects of cAMP have been attributed to the activation of protein kinase A (PKA) and subsequent changes in PKA-mediated protein expression and function [23]. In the last decade, exchange proteins directly activated by cAMP (Epac1 and Epac2) have been identified as cAMP-regulated guanine nucleotide exchange factors for Ras-like GTPases, such as Rap1 and Rap2 [25]. Epac controls a variety of cellular functions including integrin-mediated cell-adhesion [26], endothelial integrity and permeability [27], exocytosis and insulin secretion [28,29]. Epac also signals to ERK although the outcome of this particular signalling appears to depend on the cell type and specific cellular localization of Epac and their effectors [30-33]. Epac has been shown to act alone [34,35] or to either antagonize [32,36] or synergize with PKA [37,38]. Although a role of Epac in lung fibroblasts and airway smooth muscle proliferation has recently been addressed [34,35,39], the impact of both PKA and Epac on the production of inflammatory mediators in the airways is presently unknown. Here, we report on novel cAMP-driven molecular mechanisms inducing augmentation of bradykinin-induced release of IL-8 from human airway smooth muscle and we demonstrate that Epac1 and Epac2 act in concert with PKA to modulate this cellular response via signaling to the Ras-like GTPase Rap1 and ERK1/2.

## Methods

### Materials

1,4-diamino-2,3-dicyano-1, 4-bis [2-aminophenylthio]butadiene (U0126) and forskolin were purchased from Tocris (Bristol, UK). 6-Bnz-cAMP, 8-pCPT-2'-*O*-Me-cAMP, Rp-8-CPT-cAMPS, Sp-8-pCPT-2'-*O*-Me-cAMPS and 8-pCPT-2'-*O*-Me-cGMP were from BIOLOG Life Science Institute (Bremen, Germany). Fenoterol was from Boehringer Ingelheim (Ingelheim, Germany). Bradykinin, Na_3_VO_4_, aprotinin, leupeptin, pepstatin and mouse anti-β-actin antibody (A5441), peroxidase-conjugated goat anti-rabbit (A5420) and peroxidase-conjugated rabbit anti-mouse (A9044) antibodies were purchased from Sigma-Aldrich (St. Louis, MO). The anti-phospho-ERK1/2 (P-ERK1/2) (9101), anti-ERK1/2 (9102) and anti-VASP which also binds to phospho-VASP (P-VASP) (3112) were from Cell Signaling Technology (Beverly, MA). The antibodies against Rap1 (121, sc-65), Rap2 (124, sc-164) and caveolin-1 (N-20, sc-894) were purchased from Santa Cruz Biotechnology (Santa Cruz, CA), and the antibody against Rac-1 (Mab 3735) was from Millipore (Billerica, MA). The mouse monoclonal antibodies against Epac1 and Epac2 were generated and kindly provided by Dr. J. L. Bos [40]. *Clostridium difficile *toxin B-1470 was kindly provided by Drs C. von Eichel-Streiber and H. Genth. DMEM, FBS, penicillin/streptomycin solution were obtained from GIBCO-BRL Life Technologies (Paisley, UK). Alamar Blue solution was from Biosource (Camarillo, CA), the dyazo die trypan blue from Fluka Chemie (Buchs, Switzerland) and the Pierce BCA protein assay kit from Thermo Scientific (Rockford, IL). siRNA probes were purchased from Dharmacon Inc. (Lafayette, CO) and the transfection vehicle lipofectamine 2000 was from Invitrogen (Carlsbad, CA). The western lightning ECL solution was from PerkinElmer Inc. (Waltman, MA) and the IL-8 ELISA kit from Sanquin (Amsterdam, The Netherlands). All used chemicals were of analytical grade.

### Cell culture, toxin treatment, cell number and viability measurements

Human bronchial smooth muscle cell lines, immortalized by stable ectopic expression of human telomerase reverse transcriptase enzyme were used for all the experiments (hTERT-airway smooth muscle cells). The primary human bronchial smooth muscle cells used to generate these cells were prepared as described previously [41]. All procedures were approved by the human Research Ethics Board of the University of Manitoba. As described previously [42], each cell line was thoroughly characterized to passage 10 and higher. Passage 10 to 25 myocytes, grown on uncoated dishes in DMEM supplemented with antibiotics and 10% FBS, were used. Before each experiments, cells were serum deprived for one day in DMEM supplemented with antibiotics. For toxin B-1470 treatment, cells were treated for 24 hrs with 100 pg/ml toxin B-1470. Toxin-induced glucosylation of Ras-like GTPases was monitored by using a specific anti-Rac1 antibody [43], and changes in cell morphology were monitored by phase-contrast microscopy, using an Olympus IX50 microscope equipped with a digital image capture system (Color View Soft Imaging System). The toxicity of used drugs as well as their vehicle (DMSO) towards hTERT-airway smooth muscle cells was determined by an Alamar Blue assay. Briefly, cells were incubated with HBSS containing 10% vol/vol Alamar blue solution and then analyzed by fluorimetric analysis. Fluorescence derives from the conversion of Alamar blue into its reduced form by mitochondrial cytochromes and is therefore a measure of the number of cells. Viability was set as 100% in control cells. Viability of cells was also measured by resuspending cells 1:1 in the diazo dye trypan blue, which is absorbed by non viable cells, and the number of blue cells was then measured.

### Cell fractionation

Cells were lysed in 50 mM Tris (pH 7.4) supplemented with 1 mM Na_3_VO_4_, 1 mM NaF, 10 μg/ml aprotinin, 10 μg/ml leupeptin and 7 μg/ml pepstatin and then fractioned as described earlier [44]. The protein amount of all the fractions was determined using Pierce protein determination according to the manifacturer's instructions. Membrane, cytosolic and nuclear enriched fractions were subsequently used for detection of Epac1, Epac2, Rap1 and Rap2 expression.

### Silencing of Epac1 and Epac2 expression using siRNAs

Cells were transfected with siRNA probes targeted to either Epac1 or Epac2; the target sequences for human Epac1 siRNA mixture were: sense: 5'-CGUGGGAACUCAUGAGAUG-3' (J-007676-05), sense: 5'-GGACCGAGAUGCCCAAUUC-3' (J-007676-06), sense: 5'-GAGCGUCUCUUUGUUGUCA-3' (J-007676-07), sense: 5'CGUGGUACAUUAUCUGGAA-3' (J-007676-08) and for the Epac2 siRNA mixture: sense: 5'-GAACACACCUCUCAUUGAA-3' (J-009511-05), sense: 5'- GGAGAAAUAUCGACAGUAU-3' (J-009511-06), sense: 5'-GCUCAAACCUAAUGAUGUU-3' (J-009511-07), sense: 5'-CAAGUUAGCACUAGUGAAU-3' (J-009511-08). Non-silencing siRNA control was used as a control in all siRNA transfection experiments. Cells were transfected with 200 pmol of appropriate siRNA by using lipofectamine 2000 (1 mg/ml) as vehicle. 6 hrs after transfection, cells were washed with DMEM supplemented with antibiotics to reduce toxicity effects of the transfection reagent. Cells were subsequently analyzed for Epac1 and Epac2 expression, GTP-loading of Rap1 or IL-8 production.

### Activation of Rap1, phosphorylation of ERK1/2-VASP and immunoblot analysis

The amount of activated Rap1 and Rap2 was measured with the pull-down technique by using glutathione S-transferase (GST)-tagged RalGDS (Ras-binding domain of the Ral guanine nucleotide dissociation stimulator) as previously described [45]. For the measurement of the phosphorylation of ERK1/2 and VASP, cell were lysed followed by determination of the protein concentration. Equal amounts of protein (or samples) were loaded on 10-15% polyacrylamide gels and analyzed for the protein of interest by using the specific first antibody (dilution Rap1 and Rap2 1:250, P-ERK 1:1000, ERK 1:500, VASP 1:1000) and the secondary HRP-conjugated antibody (dilution 1:2000 anti-rabbit or 1:3000 anti-mouse). Protein bands were subsequently visualized on film using western lightning plus-ECL and quantified by scanning densitometry using TotalLab software (Nonlinear Dynamics, Newcastle, UK). Results were normalized for protein levels by using specific control proteins.

### IL-8 assay

The concentration of IL-8 in the culture medium was determined by ELISA according to the manifacturer's instructions (Sanquin, the Netherlands). Results were normalized for cell number according to Alamar Blue measurement. Basal IL-8 levels ranged between 0.3 and 18,8 pg/ml.

### Statistical analysis

Data were expressed as the mean ± SEM of *n *determinations. Statistical analysis was performed using the statistical software Prism. Data were compared by using an unpaired or paired two-tailed Student's t test to determine significant differences. *p *values < 0.05 were considered to be statistically significant.

## Results

### Cyclic AMP-regulated PKA and Epac augment bradykinin-induced IL-8 release from human airway smooth muscle

**Figure 1:**
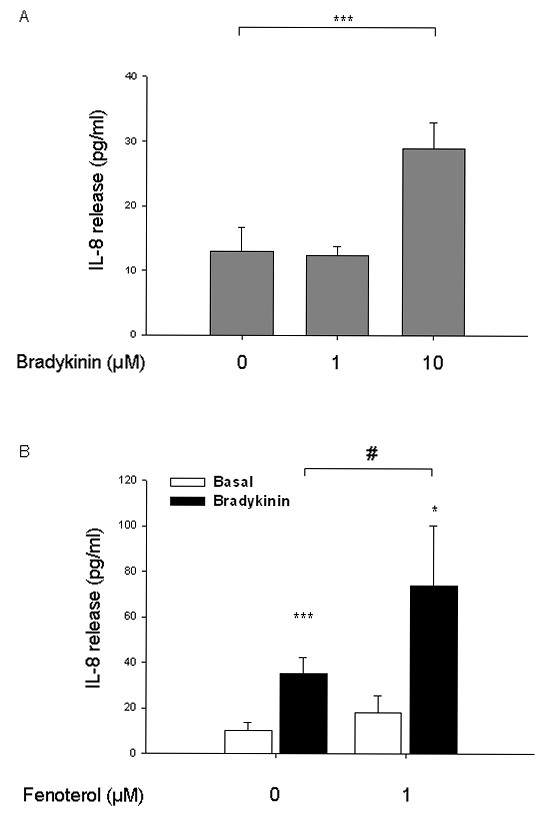
**cAMP-elevating agent fenoterol augments bradykinin-induced release of IL-8**. hTERT-airway smooth muscle cells were stimulated for 18 hrs with the indicated concentrations of bradykinin (A). Cells were incubated for 30 min without (Basal) or with 1 μM fenoterol. Then, cells were stimulated with 10 μM bradykinin for 18 hrs. IL-8 release was assessed by ELISA as described in Materials and Methods. Results are expressed as mean ± SEM of separate experiments (*n *= 3-10). *P < 0.05, ***P < 0.001 compared to unstimulated control; ^#^*P *< 0.05 compared to bradykinin-stimulated control.

Given the importance of IL-8 in airway inflammatory processes [7], we examined the role of the cAMP-elevating agent β_2_-agonist fenoterol in bradykinin-induced IL-8 release from hTERT-airway smooth muscle cells. As illustrated in Fig. 1A, bradykinin induced an increase in the release of IL-8 from the cells. The concentration of 10 μM bradykinin appeared to be most effective (~2-fold increase, *P *< 0.001) and was chosen for further experiments. The β_2_-agonist fenoterol at the concentration of 1 μM further enhanced bradykinin-induced IL-8 release of about 2 fold (*P *< 0.05), whereas it did not alter basal IL-8 production (Fig. 1B). These data suggest that bradykinin-induced IL-8 release from hTERT-airway smooth muscle cells may be augmented by cAMP signaling.

**Figure 2:**
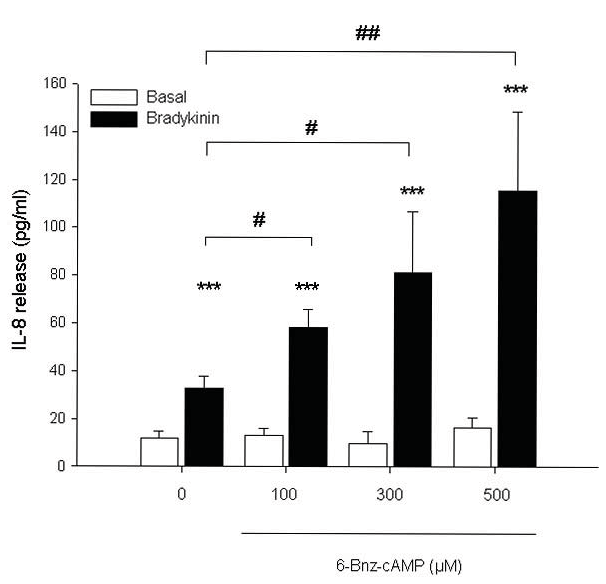
**Bradykinin-induced IL-8 release is increased by the PKA activator 6-Bnz-cAMP**. hTERT-airway smooth muscle cells were stimulated with the indicated concentrations of 6-Bnz-cAMP in the absence (Basal) or presence of 10 μM bradykinin for 18 hrs. IL-8 release was assessed by ELISA. Results are expressed as mean ± SEM of separate experiments (*n *= 3-10). ***P < 0.001 compared to unstimulated control; ^#^*P *< 0.05, ^##^*P *< 0.01 compared to bradykinin-stimulated control.

**Figure 3:**
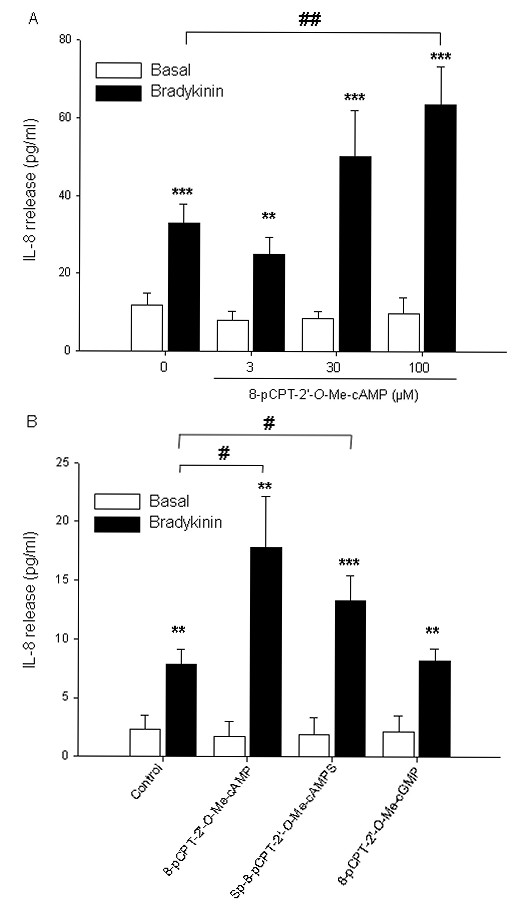
**Bradykinin-induced IL-8 release is increased by the Epac activators 8-pCPT-2'-*O*-Me-cAMP and Sp-8-pCPT-2'-*O*-Me-cAMPS**. hTERT-airway smooth muscle cells were stimulated with the indicated concentrations of 8-pCPT-2'-*O*-Me-cAMP (A) or with 100 μM of 8-pCPT-2'-*O*-Me-cAMP, Sp-8-pCPT-2'-*O*-Me-cAMPS and 8-pCPT-2'-*O*-Me-cGMP (B) in the absence (Basal) or presence of 10 μM bradykinin for 18 hrs. IL-8 release was measured by ELISA. Results are expressed as mean ± SEM of separate experiments (*n *= 3-9). **P < 0.01, ***P < 0.001 compared to unstimulated control; ^#^*P *< 0.05, ^##^*P *< 0.01 compared to bradykinin-stimulated control.

To study whether cAMP-regulated effectors PKA and Epac participate in this response, we analyzed the role of the cAMP analogs 6-Bnz-cAMP and 8-pCPT-2'-*O*-Me-cAMP known to preferentially activate PKA or Epac, respectively [46,47]. As shown in Fig. 2, direct activation of PKA by 6-Bnz-cAMP induced a concentration-dependent augmentation of bradykinin-induced IL-8 release from hTERT-airway smooth muscle cells. 500 μM 6-Bnz-cAMP induced about a 3.5-fold (*P *< 0.01) increase on bradykinin-induced IL-8 release (Fig. 2). As shown for fenoterol, 6-Bnz-cAMP did not enhance basal cellular IL-8 production at any concentration measured (Fig. 2). We report here that hTERT-airway smooth muscle cells express Epac1 and Epac2 (See later). Therefore, we also used the Epac activator 8-pCPT-2'-*O*-Me-cAMP to modulate bradykinin-induced IL-8 release. As shown in Fig. 3A, treatment of the cells with 8-pCPT-2'-*O*-Me-cAMP induced a concentration-dependent augmentation of this response. 100 μM 8-pCPT-2'-*O*-Me-cAMP increased bradykinin-induced IL-8 release by about 2-fold (*P *< 0.01) (Fig. 3A). Similar to both the β_2_-agonist fenoterol and the PKA activator 6-Bnz-cAMP, the Epac activator 8-pCPT-2'-*O*-Me-cAMP did not increase basal IL-8 production at any concentration used (Fig. 3A). To validate the data obtained with the Epac activator 8-pCPT-2'-*O*-Me-cAMP, 8-pCPT-2'-*O*-Me-cGMP, a cGMP analogue with substitutions identical to those in 8-pCPT-2'-*O*-Me-cAMP which is known to neither activate protein kinase G nor Epac [34], was used as a negative control. Moreover, Sp-8-pCPT-2'-*O*-Me-cAMPS, a phosphorothioate derivative of 8-pCPT-2'-*O*-Me-cAMP that is resistant to phosphodiesterase hydrolysis [47,48], was used as an additional Epac activator. Importantly, Sp-8-pCPT-2'-*O*-Me-cAMPS (100 μM) mimicked the effects of the Epac activator 8-pCPT-2'-*O*-Me-cAMP on bradykinin-induced IL-8 release from hTERT-airway smooth muscle cells (*P *< 0.05), whereas the negative control 8-pCPT-2'-*O*-Me-cGMP (100 μM) did not alter this response (Fig. 3B). Again, as shown for the Epac activator 8-pCPT-2'-*O*-Me-cAMP, Sp-8-pCPT-2'-*O*-Me-cAMPS and 8-pCPT-2'-*O*-Me-cGMP did not alter basal IL-8 release (Fig. 3B). Collectively, these data indicate that augmentation of bradykinin-induced IL-8 release from hTERT-airway smooth muscle cells is regulated by cAMP, likely through both PKA and Epac.

**Figure 4:**
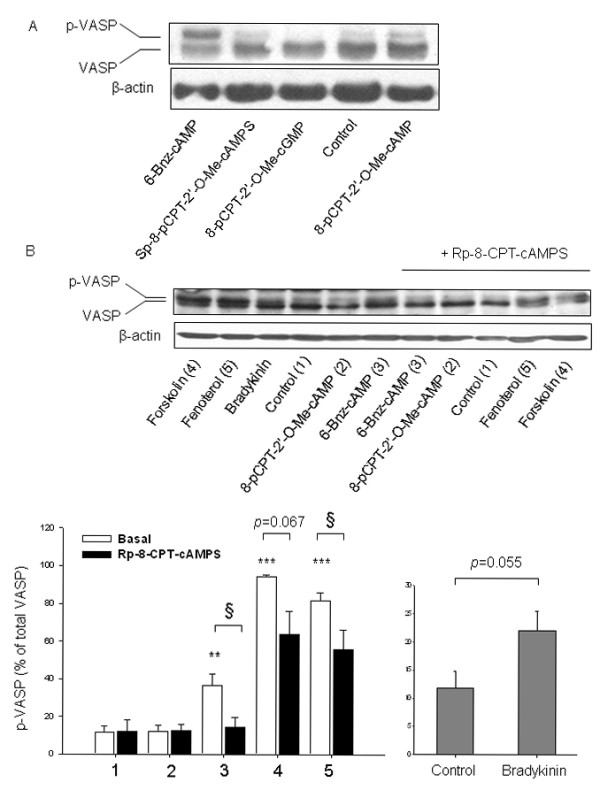
**Effects of cyclic nucleotide analogs and cAMP-elevating agents on VASP phosphorylation**. Phosphorylation of the PKA effector VASP in the absence (Control) and presence of 8-pCPT-2'-*O*-Me-cAMP, Sp-8-pCPT-2'-*O*-Me-cAMPS, 8-pCPT-2'-*O*-Me-cGMP (each 100 μM), 500 μM 6-Bnz-cAMP for 15 min was evaluated by using a VASP-specific antibody. Equal loading was verified by analysis of β-actin. Representative blots are shown (A). hTERT-airway smooth muscle cells were stimulated for 15 minutes without (Control) or with forskolin, 8-pCPT-2'-*O*-Me-cAMP (each 100 μM), 500 μM 6-Bnz-cAMP, 1 μM fenoterol and 10 μM bradykinin in the absence or presence of 100 μM Rp-8-CPT-cAMPS (B). Representative blots are shown above. Equal loading was verified by analysis of β-actin. Below are the densitometric quantifications of n = 3-6 independent experiments. Data are expressed as percentage of phospho-VASP over total VASP. **P < 0.01, ***P < 0.001 compared to unstimulated control; ^§^*P *< 0.05 compared to basal condition.

To further validate our findings, we analyzed the phosphorylation of VASP, known to be phosphorylated at Ser-157, a PKA-specific site [49], by using a VASP-specific antibody that recognizes both phospho-VASP (upper band) and total VASP (lower band). Phosphorylation of VASP was not altered by any of the Epac-related cAMP compounds being studied (each 100 μM) (Fig. 4A). In contrast, 1 μM fenoterol, 100 μM forskolin and 500 μM 6-Bnz-cAMP induced VASP phosphorylation (Fig. 4A). In addition, treatment of the cells with the pharmacological selective PKA inhibitor Rp-8-CPT-cAMPS blocked phosphorylation of VASP by 6-Bnz-cAMP (*P *< 0.05) and largely reduced VASP phosphorylation by forskolin (*P *= 0.067) and fenoterol (*P *< 0.05) (Fig. 4B). Bradykinin also induced VASP phosphorylation (*P *= 0.055) (Fig. 4B). All together, these data indicate that the cyclic nucleotides used in our study specifically activate their primary pharmacological targets PKA and Epac, and thereby induce augmentation of bradykinin-induced IL-8 release from hTERT-airway smooth muscle cells.

### Role of Ras-like GTPases in cAMP-dependent bradykinin-induced IL-8 release from human airway smooth muscle

**Figure 5:**
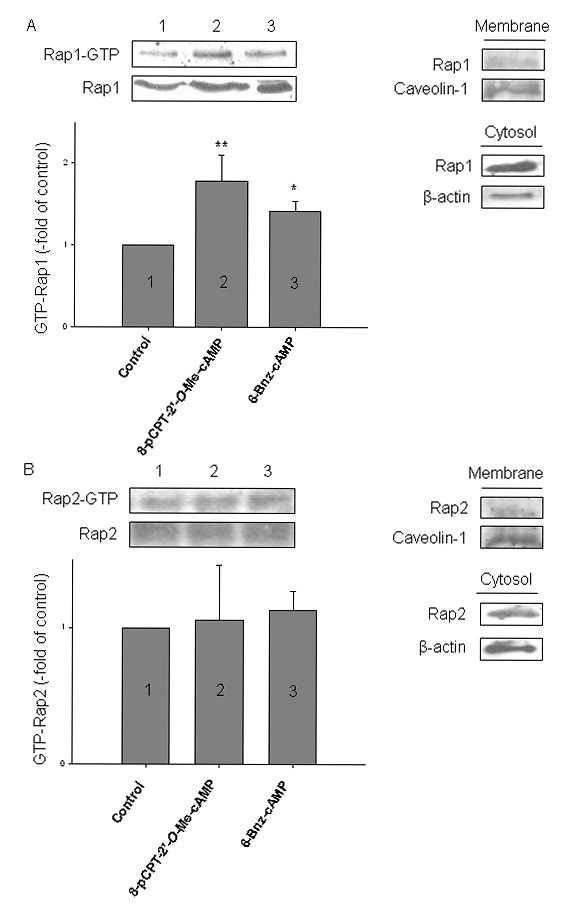
**Role of Epac and PKA in GTP-loading of Rap1 and Rap2**. hTERT-airway smooth muscle cells were fractioned as described in Material and Methods. Expression of membrane-associated or cytosolic Rap1 (A) and Rap2 (B) was evaluated and normalized to the content of the cell fraction-specific marker proteins caveolin-1 and β-actin, respectively. hTERT-airway smooth muscle cells were stimulated for 10 min without (Control) and with 100 μM 8-pCPT-2'-*O*-Me-cAMP or 500 μM 6-Bnz-cAMP. Thereafter, GTP-loaded and total Rap1 (A) or Rap2 (B) were determined as described in Material and Methods. Representative immunoblots are shown above with the respective densitometric quantifications underneath them (n = 3-5). *P < 0.05, **P < 0.01 compared to unstimulated control.

**Figure 6:**
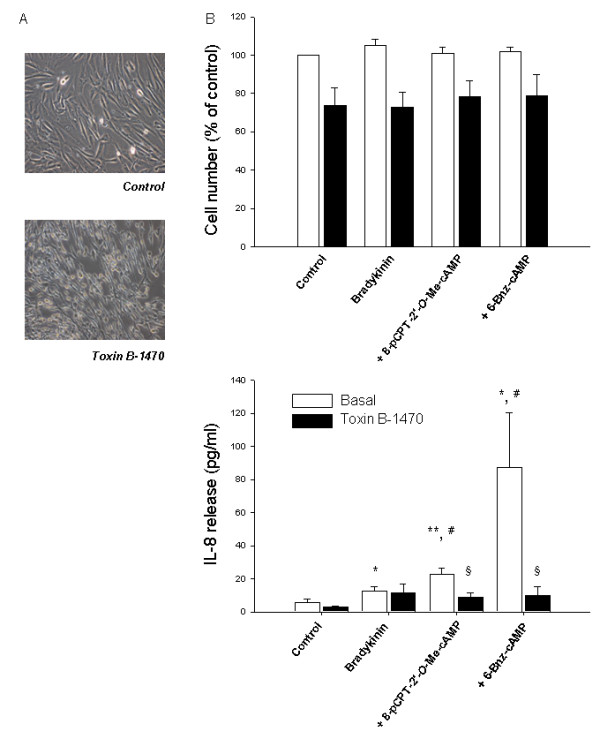
**Impact of Ras-like GTPases on bradykinin-induced IL-8 release**. hTERT cells were treated for 24 hrs without (Control) and with 100 pg/ml of *Clostridium difficile *toxin B-1470 (Toxin B-1470). Then, cell morphology was assessed by phase-contrast microscopy (A). Cell number was measured on the same cells by Alamar blue as described in Material and Methods. Data represent percentage of unstimulated control (B; upper panel). In addition, IL-8 release was measured on supernatant of cells treated with 10 μM bradykinin alone or in combination with 100 μM 8-pCPT-2'-*O*-Me-cAMP or 500 μM 6-Bnz-cAMP in the absence (Basal) or presence of 100 pg/ml Toxin B-1470 by using ELISA (B; lower panel). Results are expressed as mean ± SEM of separate experiments (*n *= 4). *P < 0.05, **P < 0.01 compared to unstimulated control; ^#^*P *< 0.05 compared to bradykinin-stimulated condition; ^§^*P *< 0.05 compared to basal condition.

PKA and Epac have been reported to modulate GTP-loading of the Ras-like GTPase Rap1 and Rap2 [45,50,51]. In hTERT-airway smooth muscle cells, Rap1 and Rap2 were both present at membrane-associated and cytosolic compartments (Fig. 5). As shown in Fig. 5A, activation of Epac by 8-pCPT-2'-*O*-Me-cAMP induced about a 2-fold increase in GTP-loading of Rap1 in hTERT-airway smooth muscle cells (*P *< 0.01). Activation of PKA by 6-Bnz-cAMP activated Rap1 by about 1,5-fold (*P *< 0.05) (Fig. 5A). In contrast, activation of Epac or PKA did not induce GTP-loading of Rap2 (Fig. 5B). To study whether activation of Ras-like GTPases by cAMP is required for the augmentation of bradykinin-induced IL-8 release, cells were treated with *Clostridium difficile *toxin B-1470 known to inactivate Ras family members, including Rap1 [52]. We analyzed cell morphology and immunoreactivity of the toxin-substrate GTPase Rac1 to monitor the functionality of toxin B-1470 [43]. Treatment of the cells with 100 pg/ml toxin B-1470 profoundly altered cell morphology, as demonstrated by the occurrence of a high number of rounded cells (Fig. 6A). Toxin B-1470 also completely abolished Rac1 immunoreactivity under any experimental condition studied (not shown). Although hTERT-airway smooth muscle cells were toxin B-1470 sensitive, toxin treatment lowered cell number only of about 20% (Fig. 6B, upper panel) and did not alter cell viability (not shown). Importantly, toxin treatment completely reversed the augmentation of bradykinin-induced IL-8 release by 8-pCPT-2'-*O*-Me-cAMP and 6-Bnz-cAMP (each *P *< 0.05), without affecting IL-8 release by bradykinin alone (Fig. 6B, lower panel). As we show that PKA and Epac induce GTP-loading of Rap1 and that inhibition of Ras-like GTPases, including Rap1, largely affect augmentation of bradykinin-induced IL-8 release by both PKA and Epac, our data point at Rap1 as an important modulator of this response.

### Role of ERK1/2 in cAMP-dependent bradykinin-induced IL-8 release from human airway smooth muscle

**Figure 7:**
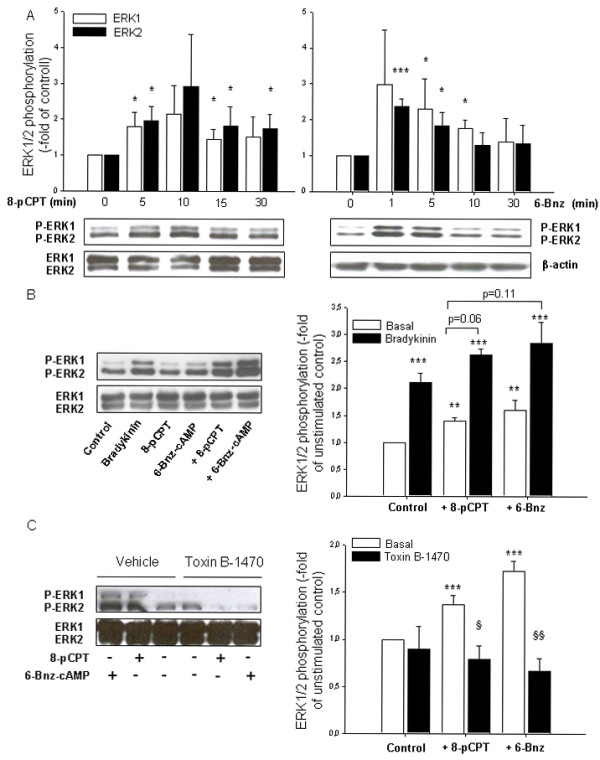
**Role of Epac and PKA in basal and bradykinin-induced ERK1/2 phosphorylation. Impact of Ras-like GTPases**. hTERT-airway smooth muscle cells were stimulated for the indicated period of time (A) or for 5 min without and with 100 μM 8-pCPT-2-*O*-Me-cAMP (8-pCPT) or 500 μM 6-Bnz-cAMP in the absence or presence of 10 μM bradykinin (10 min) (B) or 100 pg/ml *Clostridium difficile *toxin B-1470 or its vehicle (24 hrs) (C). Phosphorylated ERK1/2 (P-ERK1/2), total ERK1/2 or β-actin were detected by specific antibodies. Representative immunoblots are shown with the respective densitometric quantifications. Data are expressed as fold of ERK1/2 phosphorylation over unstimulated control and represent mean ± SEM of separate experiments (*n *= 5-7). *P < 0.05, **P < 0.01, ***P < 0.001 compared to unstimulated control; ^§^*P *< 0.05, ^§§^*P *< 0.01 compared to basal condition.

**Figure 8:**
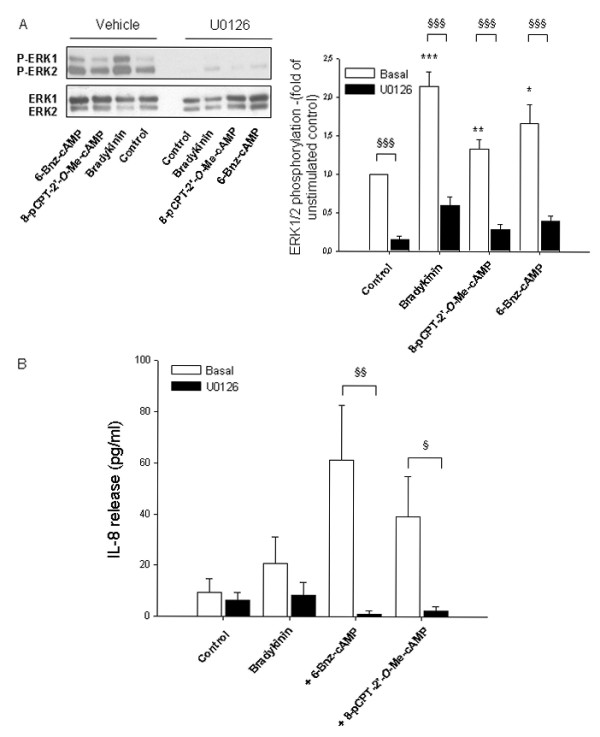
**Impact of ERK1/2 on bradykinin-induced IL-8 release and its augmentation by cAMP analogs**. Cells were pretreated for 30 min with 3 μM U0126 or vehicle before the addition of 10 μM bradykinin (15 min), 100 μM 8-pCPT-2-*O*-Me-cAMP or 500 μM 6-Bnz-cAMP (each 5 min) (A). Phosphorylated ERK1/2 (P-ERK1/2) and total ERK1/2 were detected by specific antibodies. Representative immunoblots are shown on the left with the respective densitometric quantifications on the right (n = 5). Alternatively, cells were treated with bradykinin alone or in combination with 100 μM 8-pCPT-2'-*O*-Me-cAMP or 500 μM 6-Bnz-cAMP for 18 hrs. Thereafter, IL-8 release was measured by ELISA (B). Results represent mean ± SEM of separate experiments (*n *= 3-9). *P < 0.05, **P < 0.01, ***P < 0.001 compared to unstimulated control; ^§^*P *< 0.05, ^§§^*P *< 0.01, ^§§§^*P *< 0.001 compared to basal condition.

Although the activation of ERK1/2 by Epac and PKA still remain controversial [51,53], some reports have shown that this might occur via Rap1 [32,51,54]. Current evidence also indicates that ERK1/2 regulates the expression of cytokines induced by several stimuli, including bradykinin, via activation of specific transcription factors [18,22]. To investigate whether ERK1/2 is required for the Epac- and PKA-mediated augmentation of bradykinin-induced IL-8 release from hTERT-airway smooth muscle cells, we first studied the phosphorylation of ERK1/2 in these cells by 8-pCPT-2'-*O*-Me-cAMP and 6-Bnz-cAMP. As shown in Fig. 7A, activation of Epac and PKA induced marked phosphorylation of both ERK1 and ERK2. In agreement with earlier studies [18,20], treatment with bradykinin also induced ERK1/2 phosphorylation and such stimulatory effect was further enhanced by co-stimulation with 8-pCPT-2'-*O*-Me-cAMP and 6-Bnz-cAMP (*P *= 0.06 and *P *= 0.11, respectively) (Fig. 7B). Importantly, as shown in Fig. 7C, treatment with toxin B-1470 significantly reduced ERK1/2 phosphorylation by 8-pCPT-2'-*O*-Me-cAMP (*P *< 0.05) and 6-Bnz-cAMP (*P *< 0.01). Thus, it is reasonable to assume that cAMP-dependent GTPase activation lies upstream of ERK1/2 activation in hTERT-airway smooth muscle cells. To investigate the impact of ERK1/2 on the augmentation of bradykinin-induced IL-8 release by PKA and Epac, cells were treated with U0126, a selective pharmacological inhibitor of the upstream kinase of ERK1/2, mitogen-activated protein kinase kinase (MEK) [55]. As expected, U0126 largely diminished phosphorylation of ERK1/2 under any experimental condition used (*P *< 0.001) (Fig. 8A). As illustrated in Fig. 8B, augmentation of bradykinin-induced IL-8 release by 6-Bnz-cAMP and 8-pCPT-2'-*O*-Me-cAMP was drastically impaired (*P *< 0.01 and *P *< 0.05, respectively) by MEK inhibition. As expected, treatment with U0126 also reduced bradykinin-induced IL-8 release (Fig. 8B), confirming that ERK1/2 is an important effector regulating IL-8 production. More important, our data highlight the role of ERK1/2 in augmenting bradykinin-induced IL-8 release from hTERT-airway smooth muscle cells by PKA and Epac.

### PKA and Epac cooperate to activate Rap1 and to augment bradykinin-induced IL-8 release from human airway smooth muscle

**Figure 9:**
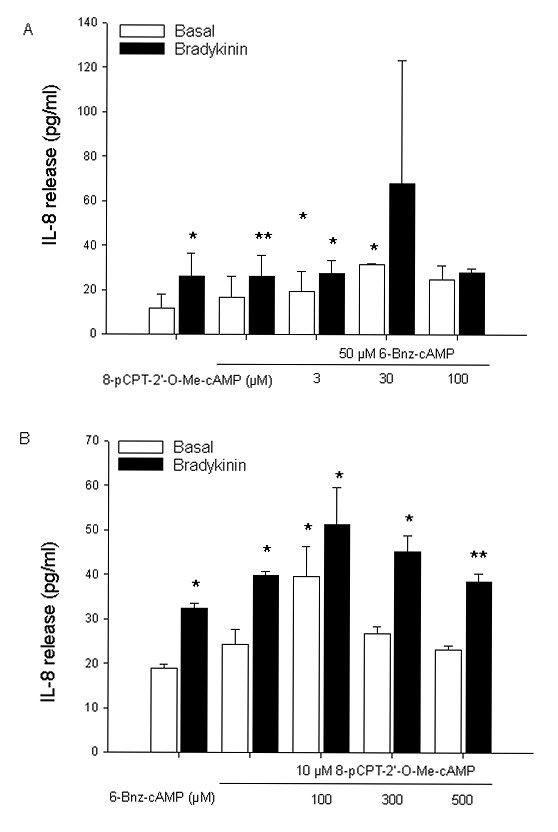
**Cooperativity of 8-pCPT-2'-*O*-Me-cAMP and 6-Bnz-cAMP on bradykinin-induced IL-8 release**. hTERT-airway smooth muscle cells were incubated with 50 μM 6-Bnz-cAMP alone or in combination with the indicated concentrations of 8-pCPT-2'-*O*-Me-cAMP (A). Alternatively, cells were stimulated with 10 μM 8-pCPT-2'-*O*-Me-cAMP alone or in combination with the indicated concentrations of 6-Bnz-cAMP (B). After that, 10 μM bradykinin was added for 18 hrs and IL-8 levels were measured by ELISA. Results represent mean ± SEM of separate experiments (*n *= 3). *P < 0.05, **P < 0.01 compared to unstimulated control.

Studies on the molecular mechanisms of cAMP-related signaling demonstrate that the classical cAMP effector PKA acts alone or in concert with the novel cAMP sensor Epac [25,56,57]. To study whether cAMP-regulated PKA and Epac might cooperate to augment bradykinin-induced IL-8 release from hTERT-airway smooth muscle cells, we stimulated the cells with 6-Bnz-cAMP in the presence of 8-pCPT-2'-*O*-Me-cAMP and *vice versa*. The effect of 50 μM 6-Bnz-cAMP on bradykinin-induced IL-8 release was modulated by 8-pCPT-2'-*O*-Me-cAMP (Fig. 9A), the most prominent effect being observed at 30 μM 8-pCPT-2'-*O*-Me-cAMP. In addition, the effects of 10 μM 8-pCPT-2'-*O*-Me-cAMP on bradykinin-induced IL-8 release were enhanced in the presence of 6-Bnz-cAMP and the maximal response was observed at 100 μM 6-Bnz-cAMP (Fig. 9B). To further validate PKA and Epac cooperative effects, we used different approaches to specifically inhibit the two cAMP-driven effectors and we studied the impact of these inhibitions on GTP-loading of Rap1 and IL-8 release from hTERT-airway smooth muscle cells.

**Figure 10:**
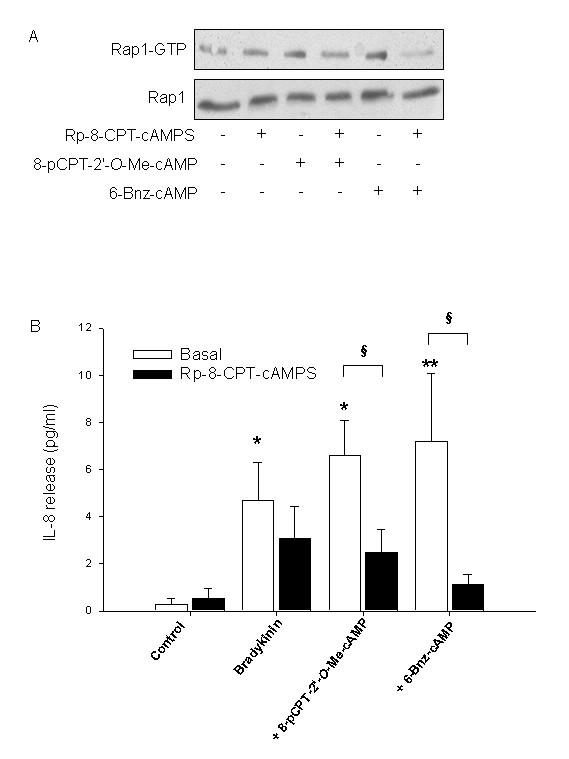
**Impact of PKA inhibition on Rap1 activation and bradykinin-induced IL-8 release**. Cells were treated for 30 min without (Basal) or with 100 μM Rp-8-CPT-cAMPS. In A, cells were first incubated with 100 μM 8-pCPT-2'-*O*-Me-cAMP or 500 μM 6-Bnz-cAMP for 5 min followed by measurement of GTP-loading of Rap1 as described in Material and Methods. Shown is a representative immunoblot. Alternatively, cells were stimulated with 10 μM bradykinin alone or in combination with 100 μM 8-pCPT-2'-*O*-Me-cAMP or 500 μM 6-Bnz-cAMP for 18 hrs (B). IL-8 release was then assessed by ELISA. Results are expressed as mean ± SEM of separate experiments (*n *= 3-7). *P < 0.05, **P < 0.01, compared to unstimulated control, ^§^*P *< 0.05 compared to basal condition.

As shown before, Rp-8-CPT-cAMPS acts as a specific inhibitor of PKA in hTERT-airway smooth muscle cells. Interestingly, treatment of cells with Rp-8-CPT-cAMPS reduced GTP-loading of Rap1 by both 8-pCPT-2'-*O*-Me-cAMP and 6-Bnz-cAMP (Fig. 10A). In addition, in the presence of Rp-8-CPT-cAMPS, augmentation of bradykinin-induced IL-8 release by the PKA activator 6-Bnz-cAMP and the Epac activator 8-pCPT-2'-*O*-Me-cAMP was largely diminished (*P *< 0.05), whereas basal and bradykinin-induced IL-8 release were not significantly altered (Fig. 10B). These data suggest that PKA and Epac pathways work in concert both at the level of Rap1 activation and the downstream production of IL-8.

**Figure 11:**
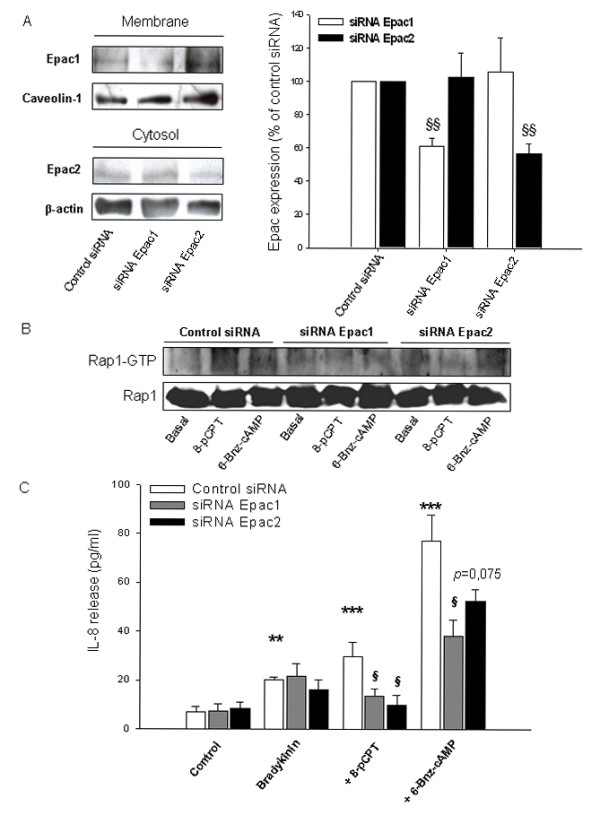
**Impact of Epac silencing on Rap1 activation and bradykinin-induced IL-8 release**. hTERT-airway smooth muscle cells were transfected for 72 hrs with control siRNA, Epac1 or Epac2 specific siRNAs (each 200 pmol). Thereafter, expression of membrane-associated Epac1 or cytosolic Epac2 was evaluated and normalized to the content of the cell fraction-specific marker proteins caveolin-1 and β-actin, respectively. Representative immunoblots are shown on the left with the respective densitometric quantifications on the right. Results are expressed as mean ± SEM of separate experiments (*n *= 5-7). Transfected cells were treated with 100 μM 8-pCPT-2'-*O*-Me-cAMP (8-pCPT) or 500 μM 6-Bnz-cAMP for 5 min and the amount of GTP-Rap1 was determined as described in Material and Methods. Shown is a representative immunoblot. In C, transfected cells were incubated with 10 μM bradykinin alone or in combination with 100 μM 8-pCPT-2'-*O*-Me-cAMP (8-pCPT) or 500 μM 6-Bnz-cAMP for 18 hrs. IL-8 release was then assessed by ELISA. Results are expressed as mean ± SEM of separate experiments (*n *= 3-7). **P < 0.01, ***P < 0.001 compared to unstimulated control; ^§^*P *< 0.05, ^§§^*P *< 0.01 compared to control siRNA.

At present, highly specific pharmacological inhibitors of individual Epac isoforms, Epac1 and Epac2, are not available [46]. Thus, to more precisely study the role of Epac1 and Epac2 in specific functions, siRNA is generally used to suppress their endogenous expression [31,34-36]. As illustrated in Fig. 11A, the siRNA approaches were effective in reducing expression of membrane-associated Epac1 and cytosolic Epac2 of about ~40% (*P *< 0.01 for Epac1 and for Epac2) leaving the expression of the cell fraction-specific marker proteins caveolin-1 and β-actin unaffected. Silencing of Epac1 and Epac2 was most efficient 72 hrs after transfection, indicating that the proteins exhibit a slow turn-over rate in hTERT-airway smooth muscle cells. As illustrated in Fig. 11B, silencing of Epac1 and Epac2 did not only reduced GTP-loading of Rap1 by 8-pCPT-2'-*O*-Me-cAMP, but also its activation by 6-Bnz-cAMP. In addition, silencing of Epac1 and Epac2 severely impaired augmentation of bradykinin-induced IL-8 release by 8-pCPT-2'-*O*-Me-cAMP (each *P *< 0.05), whereas basal and bradykinin-induced IL-8 release were again not significantly changed (Fig. 11C). Intriguingly, silencing of Epac1 also significantly reduced augmentation of bradykinin-induced IL-8 release by 6-Bnz-cAMP (*P *< 0.05) (Fig. 11C). Silencing of cellular Epac2 appeared to modestly reduce the PKA-mediated IL-8 response, although this effect was not significant (*P *= 0.075) (Fig. 11C). Taken together, these data indicate that cAMP-regulated PKA and Epac (Epac1 and Epac2) are interconnected with regard to the activation of Rap1 and the cellular production of IL-8 in hTERT-airway smooth muscle cells.

## Discussion

**Figure 12:**
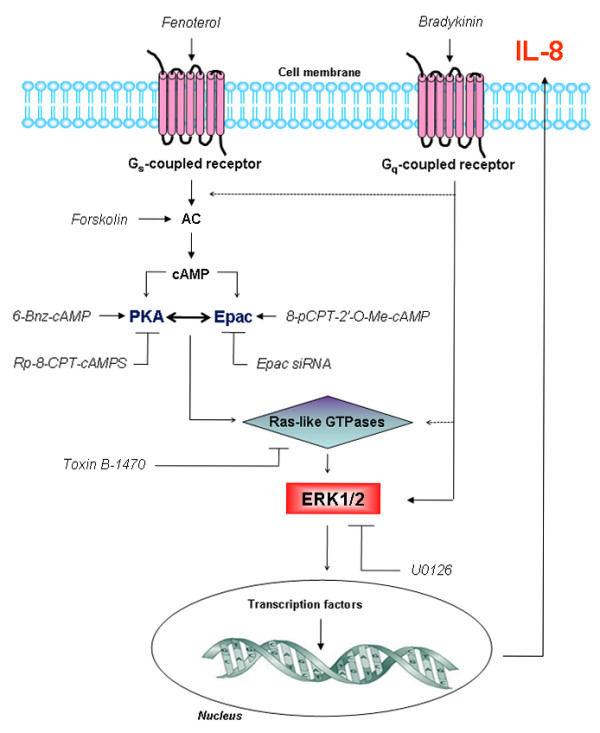
**Augmentation of bradykinin-induced IL-8 release in hTERT-airway smooth muscle cells by Epac and PKA**. Activation of ERK1/2 is mediated via different GPCRs. The β_2_-agonist fenoterol acts on G_s_-coupled receptors inducing cAMP elevation via activation of adenylyl cyclase (AC) while forskolin directly activates AC. cAMP activates two distinct cellular effectors: PKA and Epac, followed by activation of Ras-like GTPases, such as Rap1, and ERK1/2, and subsequently induction of specific transcription factors resulting in the production of IL-8. Bradykinin also elicits ERK1/2 phosphorylation most likely via activation of G_q_-coupled receptors. The dotted line represents a potential pathway which has not been fully addressed in our study. ⊥ indicates inactivation and → indicates activation, see text for further details.

Bradykinin is known to enhance the expression of several cytokines in airway smooth muscle [18,58]. cAMP-elevating agents also modulate release of cytokines from airway sources including airway smooth muscle [59]. For example, prostaglandin E_2 _(PGE_2_) was shown to increase IL-8 production in airway smooth muscle cells [6]. Interestingly, salmeterol and PGE_2 _have been reported to augment bradykinin-induced production of IL-6 by airway smooth muscle [22], but the cAMP-regulated targets responsible for this cellular response have not been identified. Here we report on novel cAMP-dependent mechanisms that augment bradykinin-induced release of IL-8 from airway smooth muscle. We demonstrate that augmentation of bradykinin-induced IL-8 production by cAMP signaling requires the cooperative action of PKA and Epac, leading subsequently to the activation of Ras-like GTPases such as Rap1 and ERK1/2 (Fig. 12).

The use of cyclic nucleotide analogs as pharmacological tools to study the specific effects of cAMP-driven signaling is now widely accepted [46]. However, studies indicated that various cyclic nucleotide analogs, including 6-Bnz-cAMP and 8-pCPT-2'-*O*-Me-cAMP might, in addition to their primary effects, also cause elevation of cAMP or cGMP upon inhibition of phosphodiesterases [47] or act upon production of cAMP hydrolysis products [48]. We did not observe indirect activation of the PKA-dependent effectors such as VASP by any of the Epac-related analogs. Moreover, phosphorylation of VASP by forskolin, fenoterol and 6-Bnz-cAMP was sensitive to the PKA inhibitor Rp-8-CPT-cAMPS. Both the PKA activator 6-Bnz-cAMP and the Epac activators used (8-pCPT-2'-*O*-Me-cAMP and Sp-8-pCPT-2'-*O*-Me-cAMPS) augmented bradykinin-induced IL-8 release in the cells, whereas no alteration of the cellular IL-8 levels was observed with the cGMP analog 8-pCPT-2'-*O*-Me-cGMP. Hence, it is reasonable to assume that PKA- and Epac-related cyclic nucleotides act via their primary pharmacological targets. Collectively, our results indicate that cAMP-dependent augmentation of bradykinin-induced IL-8 release from hTERT-airway smooth muscle cells is regulated by both PKA and Epac.

In agreement with studies in human airway smooth muscle [22], the β_2_-agonist fenoterol and the distinct PKA/Epac-related cyclic nucleotide analogs used in our studies solely alter the release of IL-8 from hTERT-airway smooth muscle in the presence of bradykinin, suggesting that this GPCR ligand might also directly affect the cAMP pathway. Previous studies have shown that bradykinin can increase intracellular levels of cAMP in airway smooth muscle via induction of cyclooxygenase and subsequent production of PGE_2 _[60]. As we observed phosphorylation of VASP by bradykinin already ≤ 15 minutes, such prostanoid-driven indirect effects may not account for the bradykinin responses observed in our study. Protein kinase C, a major downstream effector of bradykinin, has been reported to activate type II adenylyl cyclase (AC) in intact cells and to elicit activation of basal AC activity [61]. The type II AC isoform is abundantly expressed in airway smooth muscle and is activated by both Gα_s _and PKC probably leading to synergistic cAMP formation [62,63]. Moreover, PKC may cooperate in assembling the prostanoid synthetic machinery [60]. In addition, it has been reported that bradykinin inhibits approximately 60% of the total cAMP phosphodiesterase activity in guinea-pig airway smooth muscle [20]. The above mentioned mechanisms could therefore contribute to the increase of cAMP levels by bradykinin in distinct subcellular compartments and subsequently trigger the activation of PKA and Epac in airway smooth muscle.

Here we also focused on the Ras-like GTPase family members Rap1 and Rap2 as the main effectors of Epac being identified [25,64] and so far the best described in their functional association to Epac [25]. Indeed, both Rap1 and Rap2 are present in hTERT-airway smooth muscle cells in both membrane and cytosolic compartments. Interestingly, activation of PKA and Epac induced GTP-loading of Rap1 in hTERT-airway smooth muscle; both cAMP effectors did not alter basal Rap2 activity. In contrast to Epac1, activation of Rap1 by PKA has been reported to occur mostly indirectly. Evidence suggests that PKA might either activate the Rap1 exchange factor C3G (Crk Src homology domain 3) and Src [51,54,65] or inhibit the Rap1-GTPase activating protein [66]. However, it is presently unknown whether such mechanisms are operational in hTERT-airway smooth muscle. To address the role of Ras-like GTPases in bradykinin-induced IL-8 release we used the bacterial toxin B-1470. Toxin B-1470, which is produced by *C. difficile *strain 1470, inhibits exclusively the Rac protein from the Rho family and, in addition, Rap and Ral proteins from the Ras family of GTPases via glucosylation [52]. Such GTPases are important regulators of cellular adhesion and migration. Indeed, treatment with the toxin induced morphological changes and also caused cell detachment probably associated with inhibition of those GTPases. Toxin treatment only slightly reduced cell number and did not alter cell viability. Importantly, we observed a drastic reduction of bradykinin-induced IL-8 release by PKA and Epac after incubation with Toxin B-1470. Hence, our results suggest that cAMP-dependent augmentation of bradykinin-induced IL-8 requires PKA- and Epac-dependent activation of GTPases, and based on the results presented herein, Rap1 represents a very attractive candidate.

The production and release of IL-8 from airway smooth muscle upon stimulation of pro-inflammatory agonists is regulated by gene transcription and protein expression events [21]. Bradykinin has been shown to modulate the release of IL-8 generally upon activation of distinct signals including ERK1/2 [18,20]. Phosphorylation of ERK1/2 by bradykinin occurs acutely between 5-30 min in both human airway smooth muscle cells [22] and human lung fibroblasts [18]. It is generally believed that cAMP modulates transcription and protein expression [23,67], and its effects have been attributed to the phosphorylation of cAMP response element binding (CREB) protein by PKA and its subsequent binding to the CRE promoter of the specific genes [67]. Although the human IL-8 promoter does contain a CRE region, activation of CREB has not been related to the regulation of IL-8 expression in airway cells. Moreover, recent studies indicate that Epac1 also modulates gene transcription and protein expression by inducing the transcription factors CCAAT/Enhancer-binding Proteins (C/EBPs) in COS-1 cells [68]. Interestingly, both PKA and Epac have been reported to activate ERK1/2 in a cell-type specific manner [51]. Once activated, ERK1/2 signals to the nucleus, promoting transcription of genes usually associated with inflammation and proliferation. Activation of Epac and PKA in hTERT-airway smooth muscle cells increased basal ERK1/2 phosphorylation (1-30 min) and enhanced bradykinin-induced ERK1/2 phosphorylation measured after 10 min. Hence, these findings indicate that ERK1/2 activation may be an important mechanism by which β_2_-agonists augment IL-8 production in airway smooth muscle. This was confirmed by the fact that treatment with the pharmacologic inhibitor U0126 reduced the IL-8 release by bradykinin alone and even in a more pronounced way, by the combination of bradykinin with both 8-pCPT-2'-O-Me-cAMP and 6-Bnz-cAMP. The fact that toxin B-1470 treatment largely impaired ERK1/2 phosphorylation by PKA and Epac, most likely places ERK1/2 downstream of toxin B-1470-sensitive GTPases.

Previous studies in human lung fibroblasts have shown that Epac1, Epac2 and PKA act independently on distinct cellular functions [34,35]. For example, the anti-proliferative signalling properties in human lung fibroblasts have been assigned to Epac1, but not to Epac2 [34,35]. The diverse effects of Epac proteins and PKA could be explained by their different subcellular localization [69] or downstream effector availability [70,71]. Indeed, we observed that Epac isoforms Epac1 and Epac2 exhibit different cellular localization in hTERT-airway smooth muscle cells, the former being more expressed at the plasma membrane and the latter in the cytosolic fraction of the cells. However, we here demonstrate that silencing of Epac1 or Epac2 expression in hTERT-airway smooth muscle cells abolished the augmentation of bradykinin-induced IL-8 release by the Epac activator 8-pCPT-2'-*O*-Me-cAMP, and also largely diminished the enhancement of this cellular response by the PKA activator 6-Bnz-cAMP. These data point at a positive cooperativity between cAMP-regulated Epac1-Epac2 and PKA, which was confirmed by pharmacological approaches using the PKA inhibitor Rp-8-CPT-cAMPS and the combinations of the the PKA and Epac activators. Importantly, activation of Rap1 by either PKA or Epac appeared to be sensitive to inhibition of PKA by Rp-8-CPT-cAMPS or to silencing of Epac1 and Epac2 by siRNA. This results suggest that Epac and PKA work in concert to activate Rap1 and subsequently augment IL-8 release by bradykinin. Our findings might implicate that both Epac isoforms and PKA bind to the same signalling complex which are then directed to the same target(s). Indeed, distinct intracellular cAMP signaling compartments have been recently identified in primary cultures of neonatal cardiac ventriculocytes [72] and cAMP-responsive multiprotein complexes including PKA and Epac1-Epac2 seem to confer signaling specificity [73,74]. Thus, our data indicate that in airway smooth muscle both Epac1 and Epac2 act in concert with PKA to modulate pro-inflammatory signaling properties.

## Conclusion

We describe novel cAMP-dependent mechanisms to induce augmentation of bradykinin-induced IL-8 release from airway smooth muscle. Evidence is provided that cAMP-regulated Epac1 and Epac2 cooperate with PKA to induce Ras-like GTPases activation (presumably Rap1) and subsequent activation of ERK1/2. Our findings implicate that PKA, Epac1 and Epac2 exert pro-inflammatory signaling properties in human airway smooth muscle depending on the input of distinct GPCR signals. The relevance of these findings is reflected by the importance of bradykinin- and cAMP-mediated signals in airway disease pathogenesis and treatment and opens new avenues for future therapeutic intervention.

## Competing interests

The authors declare that they have no competing interests.

## Authors' contributions

SSR designed, coordinated and carried most of the work, in particular the siRNA transfections, the statistical analysis, drafted the manuscript and contributed to its design and conception. LEMK and MM performed ELISA and western blot analysis. CRSE carried out the toxin experiments. RG contributed to draft the manuscript and its design, AH and HM helped to improve the study design and the final manuscript. MS conceived of the study, participated in its study design and coordination, helped to draft and improve the manuscript. All authors read and approved the final manuscript.
